# Supplementary material for: Sticking together: independent evolution of biofilm formation in different species of staphylococci has occurred multiple times via different pathways
Source: BMC Genomics. 2024 Aug 28;25:812. doi: 10.1186/s12864-024-10719-y (PMC11350952; doi:10.1186/s12864-024-10719-y)
Supplement: Supplementary file 1 — Supplementary Material 1. [file 12864_2024_10719_MOESM1_ESM.pdf]

Supplementary information

Supplementary Table 1: Species composition and biofilm phenotype

Species (MALDI-TOF designated)	Total isolates	Isolates per biofilm category *	**	***	****
Unknown *Staphylococcus*	3	2	0	0	1
Staphylococcus auricularis	2	2	0	0	0
Staphylococcus capitis	19	13	2	1	3
Staphylococcus caprae	2	0	0	1	1
Staphylococcus chromogenes	3	0	0	0	3
Staphylococcus cohnii	1	0	0	0	1
Staphylococcus epidermidis	193	62	53	26	52
Staphylococcus equorum	1	1	0	0	0
Staphylococcus haemolyticus	44	35	2	5	2
Staphylococcus hominis	44	31	11	2	0
Staphylococcus lugdunensis	9	5	2	2	0
Staphylococcus pasteuri	3	2	1	0	0
Staphylococcus pettenkferi	1	1	0	0	0
Staphylococcus saprophyticus	22	6	6	5	5
Staphylococcus sciuri	5	1	0	3	1
Staphylococcus simulans	9	0	3	3	3
Staphylococcus succinus	1	1	0	0	0
Staphylococcus vitulinus	3	2	0	0	1
Staphylococcus warneri	18	11	5	1	1
Staphylococcus xylosus	2	1	0	0	1

Assessment of clonal nature of isolates using Snippy:

Fastq files were processed using Snippy and Snippy-core [v4.4.3] and then the core alignment was used to infer a Maximum Likelihood tree using IQTree [v1.6.12]. Snpdist [v0.6.3] was used to compare number of snps amongst the core genome. Reference strain RP62A (NC_002976.3) was used for *S. epidermidis* and *S. haemolyticus* NCTC 11042 (NZ_UHDN01000001.1).

Supplementary Table 2: Snippy-core matrix showing SNPs different from the reference genome (*S. epidermidis* RP62A) in *S. epidermidis* isolates. Length – length of the reference. Aligned – sites aligned to. Unaligned – Sites unaligned. Variant – Number of sites different from reference. Het – heterozygous or poor quality genotype. Masked – sites masked in reference. Lowcov – low coverage sites.

ID	LENGTH	ALIGNED	UNALIGNED	VARIANT	HET	MASKED	LOWCOV
15TB0532_S110	2616530	2265028	329210	11698	342	0	21950
15TB0533_S107	2616530	2279618	328046	11775	850	0	8016
15TB0706_S1	2616530	2294668	296088	12042	564	0	25210
15TB0707_S155	2616530	2334903	271979	10065	1215	0	8433
15TB0708_S8	2616530	2331986	271349	10029	914	0	12281
15TB0709_S14	2616530	2282576	326347	10362	1187	0	6420
15TB0714_S12	2616530	2284627	308680	11998	696	0	22527
15TB0720_S6	2616530	2312027	295843	6398	919	0	7741
15TB0722_S87	2616530	2318966	289656	9569	1355	0	6553
15TB0723_S2	2616530	2303033	280733	9385	1081	0	31683
15TB0724_S16	2616530	2243641	363305	12401	409	0	9175
15TB0725_S17	2616530	2293607	312936	10426	936	0	9051
15TB0726_S3	2616530	2213406	351903	17088	315	0	50906
15TB0727_S4	2616530	2258671	314227	16958	758	0	42874
15TB0733_S163	2616530	2329121	279081	10167	1465	0	6863
15TB0734_S159	2616530	2216271	368286	48944	981	0	30992
15TB0736_S162	2616530	2312317	294242	8907	738	0	9233
15TB0739_S167	2616530	2307572	292871	8854	827	0	15260
15TB0743_S11	2616530	2314334	282853	8855	882	0	18461
15TB0750_S150	2616530	2267746	292889	10324	1103	0	54792
15TB0752_S9	2616530	2274783	327821	10097	328	0	13598
15TB0753_S10	2616530	2298738	292440	8654	751	0	24601
15TB0754_S157	2616530	2232254	334741	16394	414	0	49121
15TB0756_S20	2616530	2312106	293733	8676	937	0	9754
15TB0763_S21	2616530	2260483	345593	8518	1058	0	9396
15TB0768_S22	2616530	2309041	297056	5697	1157	0	9276
15TB0769_S152	2616530	2311163	292406	5674	702	0	12259
15TB0770_S23	2616530	2310032	293262	8490	1038	0	12198
15TB0771_S24	2616530	2309809	293948	8487	823	0	11950
15TB0772_S25	2616530	2244620	355031	26007	1098	0	15781
15TB0778_S154	2616530	2310425	292989	8473	673	0	12443
15TB0783_S32	2616530	2270712	333218	9957	116	0	12484
15TB0785_S33	2616530	2313413	293324	8435	898	0	8895
15TB0789_S35	2616530	2280124	326679	9889	318	0	9409

15TB0791_S104	2616530	2286756	321058	10308	707	0	8009
15TB0796_S37	2616530	2212881	388428	45082	538	0	14683
15TB0799_S38	2616530	2300076	310292	10277	614	0	5548
15TB0802_S40	2616530	2263927	344884	9009	408	0	7311
15TB0805_S41	2616530	2300862	305866	9034	923	0	8879
15TB0808_S42	2616530	2214746	388771	45052	692	0	12321
15TB0812_S43	2616530	2278615	330325	8999	755	0	6835
15TB0814_S82	2616530	2174193	331820	9138	203	0	110314
15TB0820_S83	2616530	2102870	371252	41380	536	0	141872
15TB0825_S85	2616530	2231973	346266	9389	104	0	38187
15TB0830_S10	2616530	2214313	384760	40888	532	0	16925
15TB0832_S85	2616530	2223575	381436	40279	683	0	10836
15TB0833_S88	2616530	2275396	326452	9355	218	0	14464
15TB0838_S103	2616530	2227736	376695	41242	901	0	11198
15TB0842_S142	2616530	2276521	331181	10217	358	0	8470
15TB0844_S144	2616530	2285294	324747	8993	893	0	5596
15TB0845_S145	2616530	2277793	329992	10364	525	0	8220
15TB0846_S146	2616530	2266961	339689	9284	611	0	9269
15TB0847_S139	2616530	2240207	364470	41045	993	0	10860
15TB0853_S2	2616530	2254872	342752	22555	307	0	18599
15TB0854_S92	2616530	2307223	290393	8244	255	0	18659
15TB0859_S124	2616530	2220600	385201	24385	306	0	10423
15TB0860_S125	2616530	2315184	295639	4084	459	0	5248
15TB0863_S113	2616530	2273992	331770	9491	806	0	9962
15TB0868_S128	2616530	2307478	303323	8237	473	0	5256
15TB0870_S108	2616530	2219526	385667	24455	365	0	10972
15TB0872_S121	2616530	2309347	302363	8221	537	0	4283
15TB0873_S130	2616530	2276579	333171	9341	815	0	5965
15TB0874_S131	2616530	2279204	328017	8375	307	0	9002
15TB0877_S134	2616530	2281531	327557	8771	606	0	6836
15TB0878_S148	2616530	2271354	336127	9060	476	0	8573
15TB0879_S135	2616530	2267779	331091	9008	117	0	17543
15TB0880_S109	2616530	2322260	288439	8968	759	0	5072
15TB0883_S137	2616530	2283565	325443	8933	532	0	6990
15TB0888_S94	2616530	2200718	366362	40538	726	0	48724
15TB0899_S100	2616530	2272862	332068	10100	403	0	11197
15TB0902_S147	2616530	2277084	322744	9204	362	0	16340
15TB0904_S30	2616530	2278632	329460	8357	437	0	8001
15TB0910_S114	2616530	2275231	333718	10142	368	0	7213
15TB0912_S82	2616530	2278839	329843	8351	1331	0	6517
15TB0913_S150	2616530	2243490	335827	7026	745	0	36468
15TB0914_S151	2616530	2268039	343415	10505	410	0	4666
15TB0916_S107	2616530	2333988	274475	7639	920	0	7147
15TB0925_S158	2616530	2298995	310815	8196	1011	0	5709
15TB0926_S79	2616530	2285645	320037	8175	943	0	9905
15TB0927_S159	2616530	2301379	308576	8931	738	0	5837

15TB0930_S53	2616530	2280276	321620	9462	309	0	14325
15TB0931_S54	2616530	2278777	317244	9106	415	0	20094
15TB0932_S55	2616530	2291155	318823	7407	672	0	5880
15TB0936_S29	2616530	2310491	294357	7641	848	0	10834
15TB0942_S92	2616530	2291558	317109	8333	758	0	7105
15TB0945_S63	2616530	2261723	341314	39583	484	0	13009
15TB0951_S50	2616530	2298473	306591	7768	941	0	10525
15TB0955_S47	2616530	2223551	382819	39793	734	0	9426
15TB0957_S131	2616530	2306808	300447	7529	1186	0	8089
15TB0958_S114	2616530	2275811	328344	14202	361	0	12014
15TB0971_S67	2616530	2236517	358970	39044	483	0	20560
15TB0972_S68	2616530	2238274	308134	7871	227	0	69895
15TB0979_S70	2616530	2082061	369474	37155	126	0	164869
15TB0986_S72	2616530	2202092	356079	16305	108	0	58251
15TB0987_S73	2616530	2173564	376958	38336	341	0	65667
15TB0988_S74	2616530	2333251	273386	7341	1156	0	8737
15TB0994_S111	2616530	2288439	310054	8520	521	0	17516
15TB0995_S75	2616530	2284950	308874	8599	387	0	22319
15TB0998_S77	2616530	2262031	328431	8376	346	0	25722
15TB0999_S78	2616530	2260792	328456	8352	279	0	27003
16TB0555_S167	2616530	2240563	346610	37457	1003	0	28354
16TB0559_S7	2616530	2280787	324198	8375	479	0	11066
16TB0560_S164	2616530	2278880	327017	8390	326	0	10307
16TB0567_S161	2616530	2288804	321530	8993	738	0	5458
16TB0568_S163	2616530	2286728	321274	8973	523	0	8005
16TB0569_S162	2616530	2276090	316514	7987	273	0	23653
16TB0571_S165	2616530	2320418	289554	8616	658	0	5900
16TB0577_S127	2616530	2194756	405070	38359	343	0	16361
16TB0579_S86	2616530	2213029	385265	37531	945	0	17291
16TB0594_S128	2616530	2302591	305075	7435	945	0	7919
16TB0597_S177	2616530	2221407	382558	34605	512	0	12053
16TB0598_S93	2616530	2205135	398464	38441	825	0	12106
16TB0599_S80	2616530	2312160	288060	7302	915	0	15395
16TB0600_S178	2616530	2263413	345353	7603	242	0	7522
16TB0604_S33	2616530	2278960	329735	8817	782	0	7053
16TB0605_S117	2616530	2326297	275974	7278	822	0	13437
16TB0606_S180	2616530	2299772	308967	8273	277	0	7514
16TB0609_S97	2616530	2265552	344683	8819	1262	0	5033
16TB0610_S182	2616530	2222325	383078	37436	766	0	10361
16TB0612_S183	2616530	2237995	374623	9457	801	0	3111
16TB0613_S88	2616530	2246216	358776	38123	1110	0	10428
16TB0616_S136	2616530	2315195	290456	8575	582	0	10297
16TB0617_S137	2616530	2314854	290716	8591	545	0	10415
16TB0618_S94	2616530	2211364	392114	38321	1087	0	11965
16TB0619_S138	2616530	2288302	317528	7664	572	0	10128
16TB0621_S140	2616530	2291072	317182	10859	445	0	7831

16TB0622_S141	2616530	2279177	320789	9090	612	0	15952
16TB0626_S132	2616530	2290827	317796	8959	499	0	7408
16TB0627_S112	2616530	2266688	337810	7951	364	0	11668
R26951_S5	2616530	2277172	316867	8341	253	0	22238
R34108_S40	2616530	2307224	271714	8127	918	0	36674
R34109_S32	2616530	2330765	268075	8169	1804	0	15886
R34110_S35	2616530	1981734	281738	7225	420	0	352638
R34111B_S22	2616530	2332156	271955	7319	1043	0	11376
R360752_S36	2616530	2278858	286126	6621	528	0	51018
R_Dean_1	2616530	1921847	337797	6126	251	0	356635
R_Dean_12	2616530	2005992	406115	31450	483	0	203940
R_Dean_17	2616530	1994455	350534	8500	312	0	271229
R_Dean_25	2616530	1961599	353166	6958	97	0	301668
R_Dean_27	2616530	2040876	341090	6005	350	0	234214
R_Dean_30	2616530	2073166	360740	7255	200	0	182424
R_Dean_31	2616530	2049059	348838	7118	153	0	218480
R_Dean_36	2616530	2099828	341634	6291	175	0	174893
R_Dean_39	2616530	2020357	376757	6642	58	0	219358
R_Dean_41	2616530	1927972	361052	6717	52	0	327454
R_Dean_44	2616530	2020100	353544	7736	145	0	242741
R_Dean_45	2616530	2043921	392544	30164	439	0	179626
R_Dean_48	2616530	2084371	356748	6214	318	0	175093
R_Dean_5	2616530	2017577	408348	30020	525	0	190080
R_Dean_66	2616530	2065441	365667	5192	274	0	185148
R_Dean_7	2616530	1970378	343932	6170	303	0	301917
R_Dean_77	2616530	2109301	357838	7517	322	0	149069
R_Dean_84	2616530	2065491	329185	8440	514	0	221340
R_Dean_86	2616530	2027334	409643	29115	490	0	179063
R_Dean_9	2616530	2033986	336929	6113	293	0	245322
R_Dean_90	2616530	1973007	367231	6642	117	0	276175
R_Dean_91	2616530	1998801	372832	6820	124	0	244773
R_Dean_92	2616530	2033992	345337	6624	153	0	237048
YEAC1_S48	2616530	2261486	341712	6694	541	0	12791
Reference	2616530	2616530	0	0	0	0	0

Supplementary Table 3: Snippy-core matrix showing SNPs different from the reference genome (*S. haemolyticus* NCTC 11042) in *S. haemolyticus* isolates. Length – length of the reference. Aligned – sites aligned to. Unaligned – Sites unaligned. Variant – Number of sites different from reference. Het – heterozygous or poor quality genotype. Masked – sites masked in reference. Lowcov – low coverage sites.

ID	LENGTH	ALIGNED	UNALIGNED	VARIANT	HET	MASKED	LOWCOV
15TB0710_S37	2515409	2107870	381937	51960	504	0	25098
15TB0737_S160	2515409	2172071	255080	8347	106	0	88152
15TB0748_S46	2515409	2128265	375147	7461	363	0	11634
15TB0762_S45	2515409	2175968	324298	36263	884	0	14259
15TB0807_S39	2515409	2306483	195520	4959	705	0	12701
15TB0826_S86	2515409	2193102	293502	18604	167	0	28638
15TB0834_S91	2515409	2274408	233557	5905	1146	0	6298
15TB0836_S89	2515409	2195610	299172	9799	284	0	20343
15TB0840_S3	2515409	2264944	237757	7750	869	0	11839
15TB0848_S140	2515409	2270468	236306	5081	385	0	8250
15TB0849_S72	2515409	2287868	217302	5330	621	0	9618
15TB0857_S122	2515409	2271794	231368	7693	378	0	11869
15TB0865_S26	2515409	2266360	232437	7641	286	0	16326
15TB0867_S31	2515409	2258004	232396	7639	565	0	24444
15TB0869_S129	2515409	2264581	236563	7645	851	0	13414
15TB0875_S36	2515409	2290761	207868	5329	481	0	16299
15TB0876_S133	2515409	2265573	226440	5873	660	0	22736
15TB0881_S40	2515409	2229453	266110	9575	539	0	19307
15TB0918_S17	2515409	2259184	239571	7496	570	0	16084
15TB0924_S157	2515409	2265053	238221	5288	492	0	11643
15TB0929_S160	2515409	2210228	290581	9544	335	0	14265
15TB0948_S61	2515409	2200349	251146	5869	47	0	63867
15TB0984_S105	2515409	2259481	242565	7401	134	0	13229
15TB1000_S73	2515409	2271410	232455	5633	404	0	11140
16TB0563_S144	2515409	2271923	232044	7409	439	0	11003
16TB0564_S5	2515409	2267201	232997	7353	319	0	14892
R360741_S30	2515409	1694208	276102	5153	47	0	545052
R360742B_S45	2515409	2251156	235529	5308	136	0	28588
R_Dean_24	2515409	2091997	254462	3888	250	0	168700
R_Dean_43	2515409	1982368	349212	25787	192	0	183637
R_Dean_51	2515409	2067658	264454	5643	106	0	183191
R_Dean_68	2515409	2120925	258823	3817	301	0	135360
R_Dean_85	2515409	2131356	260023	4081	419	0	123611
R_Dean_94	2515409	2076344	250143	4094	677	0	188245
YEAC2_S44	2515409	2159416	301633	24922	226	0	54134
Reference	2515409	2515409	0	0	0	0	0

Supplementary Table 4: Top 100 outputs for significant Pfam domains

Pfam domain	baseMean[a]	log2 Fold Change[b]	Standard error[c]	stat[d]	P value	Adjusted P value
Apc3	7.21779	0.41153	0.07554	5.44807	5.09E-08	8.65E-05
HYR	2.21602	0.43653	0.08514	5.12748	2.94E-07	0.000249
Bre5	1.79488	0.43034	0.08651	4.97429	6.55E-07	0.00036
Fer4	2.62659	-0.42478	0.08627	-4.92390	8.48E-07	0.00036
G5	10.34756	0.40584	0.08379	4.84376	1.27E-06	0.000433
Chitin_synth_2	1.61552	0.41303	0.08868	4.65729	3.20E-06	0.00088
SdrG_C_C	3.11266	0.38897	0.08398	4.63153	3.63E-06	0.00088
Thi4	2.25859	-0.39845	0.08790	-4.53328	5.81E-06	0.00123
Y_Y_Y	2.56733	0.38010	0.08486	4.47886	7.50E-06	0.00142
HEAT	1.93614	-0.38199	0.08774	-4.35382	1.34E-05	0.00227
A2M_N	1.65023	0.36604	0.08679	4.21737	2.47E-05	0.00343
DNA_methylase	1.73501	-0.37043	0.08783	-4.21749	2.47E-05	0.00343
He_PIG	5.29161	0.34977	0.08321	4.20367	2.63E-05	0.00343
Amidohydro_3	2.62556	0.35519	0.08733	4.06720	4.76E-05	0.00577
Big_3	1.58708	0.35067	0.08710	4.02591	5.68E-05	0.00642
Rib	5.58124	0.34998	0.08797	3.97847	6.94E-05	0.00736
EamA	6.83724	-0.28577	0.07233	-3.95095	7.78E-05	0.00777
Abhydrolase_2	1.73488	-0.32991	0.08791	-3.75286	0.000175	0.0156
PKD	2.06183	0.32163	0.08544	3.76455	0.000167	0.0156
BcrAD_BadFG	1.55211	-0.32607	0.08788	-3.71053	0.000207	0.0176
His_biosynth	2.38711	0.31326	0.08676	3.61075	0.000305	0.0247
ABG_transport	1.91528	-0.29610	0.08778	-3.37313	0.000743	0.0574
Gram_pos_anchor	3.18956	0.27819	0.08358	3.32824	0.000874	0.0639
HTH_Mga	2.10600	0.29325	0.08835	3.31905	0.000903	0.0639
DNA_pol3_gamma3	1.46783	-0.28693	0.08774	-3.27009	0.00108	0.0702
KorB	2.09479	0.28689	0.08767	3.27233	0.00107	0.0702
Amino_oxidase	3.04457	-0.26847	0.08466	-3.17133	0.00152	0.0853
Collagen	2.88354	0.25403	0.08026	3.16511	0.00155	0.0853
HI0933_like	13.28017	0.17711	0.05532	3.20164	0.00137	0.0853
MarR	7.01608	-0.22202	0.07058	-3.14553	0.00166	0.0853
NAD_binding_4	3.60155	-0.27173	0.08621	-3.15176	0.00162	0.0853
NmrA	3.70011	-0.25834	0.08196	-3.15192	0.00162	0.0853
Sulfate_transp	2.29662	-0.27828	0.08733	-3.18667	0.00144	0.0853
Y1_Tnp	2.33946	0.26859	0.08675	3.09606	0.00196	0.0971
Zeta_toxin	1.86479	-0.26914	0.08710	-3.09004	0.002	0.0971
Flavodoxin_2	3.20046	-0.25899	0.08410	-3.07937	0.00207	0.0978
Amidohydro_2	1.74362	-0.26987	0.08802	-3.06593	0.00217	0.0996
Str_synth	2.03754	0.26194	0.08782	2.98256	0.00286	0.128
Gly_radical	2.38824	0.24705	0.08674	2.84814	0.0044	0.191
Aminotran_5	8.01658	0.18675	0.06601	2.82905	0.00467	0.194
DAO	31.15039	0.11248	0.03978	2.82761	0.00469	0.194
IncA	2.90806	0.24535	0.08734	2.80902	0.00497	0.201
GTF2I	1.48010	0.23979	0.08649	2.77247	0.00556	0.22

Lipoprotein_Ltp	3.92379	0.21675	0.08056	2.69071	0.00713	0.275
FctA	1.59361	-0.22450	0.08744	-2.56740	0.0102	0.382
Glyoxalase	5.58347	-0.20082	0.07877	-2.54955	0.0108	0.382
Ndr	1.75089	0.22564	0.08845	2.55110	0.0107	0.382
Z1	1.30747	-0.22362	0.08747	-2.55645	0.0106	0.382
Peptidase_A24	1.99603	0.22275	0.08794	2.53301	0.0113	0.392
Gate	6.00348	0.18194	0.07222	2.51936	0.0118	0.399
ACT	4.30250	0.19432	0.07885	2.46453	0.0137	0.409
FMN_red	2.82824	-0.21074	0.08526	-2.47160	0.0135	0.409
Hexapep	15.66674	-0.12986	0.05269	-2.46472	0.0137	0.409
Methyltransf_4	3.07418	0.20919	0.08374	2.49811	0.0125	0.409
Mob_Pre	2.01137	0.21718	0.08782	2.47293	0.0134	0.409
NuiA	1.09365	0.21611	0.08713	2.48049	0.0131	0.409
Patatin	1.55021	-0.21770	0.08804	-2.47284	0.0134	0.409
YSIRK_signal	8.72584	0.15715	0.06458	2.43359	0.015	0.438
AlaDh_PNT_C	2.75057	0.20508	0.08522	2.40650	0.0161	0.464
HxlR	2.00005	-0.21017	0.08773	-2.39547	0.0166	0.47
Acetyltransf_1	18.50956	-0.12277	0.05301	-2.31624	0.0205	0.527
DM13	1.99580	0.20496	0.08787	2.33256	0.0197	0.527
GA	28.07883	0.19923	0.08599	2.31700	0.0205	0.527
Ldh_1_C	3.44001	0.19102	0.08218	2.32427	0.0201	0.527
Mac	1.50218	-0.20371	0.08793	-2.31661	0.0205	0.527
PRA.CH	1.69433	0.20752	0.08850	2.34474	0.019	0.527
TMP.TENI	3.99749	0.18397	0.07959	2.31150	0.0208	0.527
PPR	1.38293	-0.20140	0.08741	-2.30410	0.0212	0.53
FIVAR	41.04384	0.19031	0.08351	2.27887	0.0227	0.558
Abi	5.69711	-0.16809	0.07416	-2.26673	0.0234	0.56
Peptidase_M18	2.08070	0.19883	0.08766	2.26822	0.0233	0.56
NADH.G_4Fe.4S_3	1.56154	-0.19853	0.08803	-2.25522	0.0241	0.569
His_kinase	2.28043	0.19515	0.08705	2.24179	0.025	0.581
NMO	3.56269	-0.18108	0.08206	-2.20670	0.0273	0.627
Terminase_4	1.43280	0.19495	0.08856	2.20140	0.0277	0.627
Bac_luciferase	4.91876	-0.16502	0.07709	-2.14054	0.0323	0.722
HisG	1.65996	0.18619	0.08852	2.10349	0.0354	0.728
HTH_17	1.51178	0.18607	0.08854	2.10148	0.0356	0.728
IGPD	1.65996	0.18619	0.08852	2.10349	0.0354	0.728
Molybdop_Fe4S4	1.54617	-0.18508	0.08804	-2.10224	0.0355	0.728
Ribosomal_S4e	1.67540	0.17419	0.08276	2.10488	0.0353	0.728
SBF	1.55241	-0.18588	0.08799	-2.11261	0.0346	0.728
Ubie_methyltran	2.80073	-0.18110	0.08551	-2.11782	0.0342	0.728
CPDase	1.36085	-0.18257	0.08754	-2.08561	0.037	0.747
Peptidase_M4_C	2.28903	0.18117	0.08703	2.08157	0.0374	0.747
GAF	1.42696	0.18185	0.08848	2.05520	0.0399	0.752
Methyltransf_15	1.35451	-0.18095	0.08772	-2.06277	0.0391	0.752
Peptidase_U35	1.27255	0.18199	0.08819	2.06367	0.039	0.752
PFL	1.64459	0.18207	0.08853	2.05671	0.0397	0.752

PRA.PH	1.66288	0.18279	0.08851	2.06519	0.0389	0.752
Competence_A	2.33251	0.17716	0.08683	2.04028	0.0413	0.771
Acid_phosphat_B	1.55906	0.18008	0.08856	2.03339	0.042	0.775
ATP.grasp_3	4.92616	-0.15601	0.07695	-2.02752	0.0426	0.778
Histidinol_dh	1.69805	0.17892	0.08847	2.02243	0.0431	0.779
HSP20	1.50674	-0.17724	0.08796	-2.01494	0.0439	0.785
PAF.AH_p_II	1.22877	-0.17507	0.08729	-2.00564	0.0449	0.794
BioW	1.53722	0.17722	0.08856	2.00114	0.0454	0.794
Condensation	2.28126	0.17159	0.08705	1.97111	0.0487	0.836
ICMT	1.68156	0.17431	0.08849	1.96985	0.0489	0.836

Metrics produced as standard DESeq2 outputs (Love, Huber, & Anders, 2014). **a**: average of the normalized count values, divided by size factors and taken over all samples. **b**: how much the domain presence has changed between the groups studied. Reported on a logarithmic scale (base 2). **c**: standard error for the log2 Fold Change estimate. **d**: Wald statistic value for the Pfam domain tested. **e**: Wald test p-value. **f**: Benjamini-Hochberg adjusted p-value, used for significance cut-off.

Supplementary Figure 1: Decision tree used for machine learning

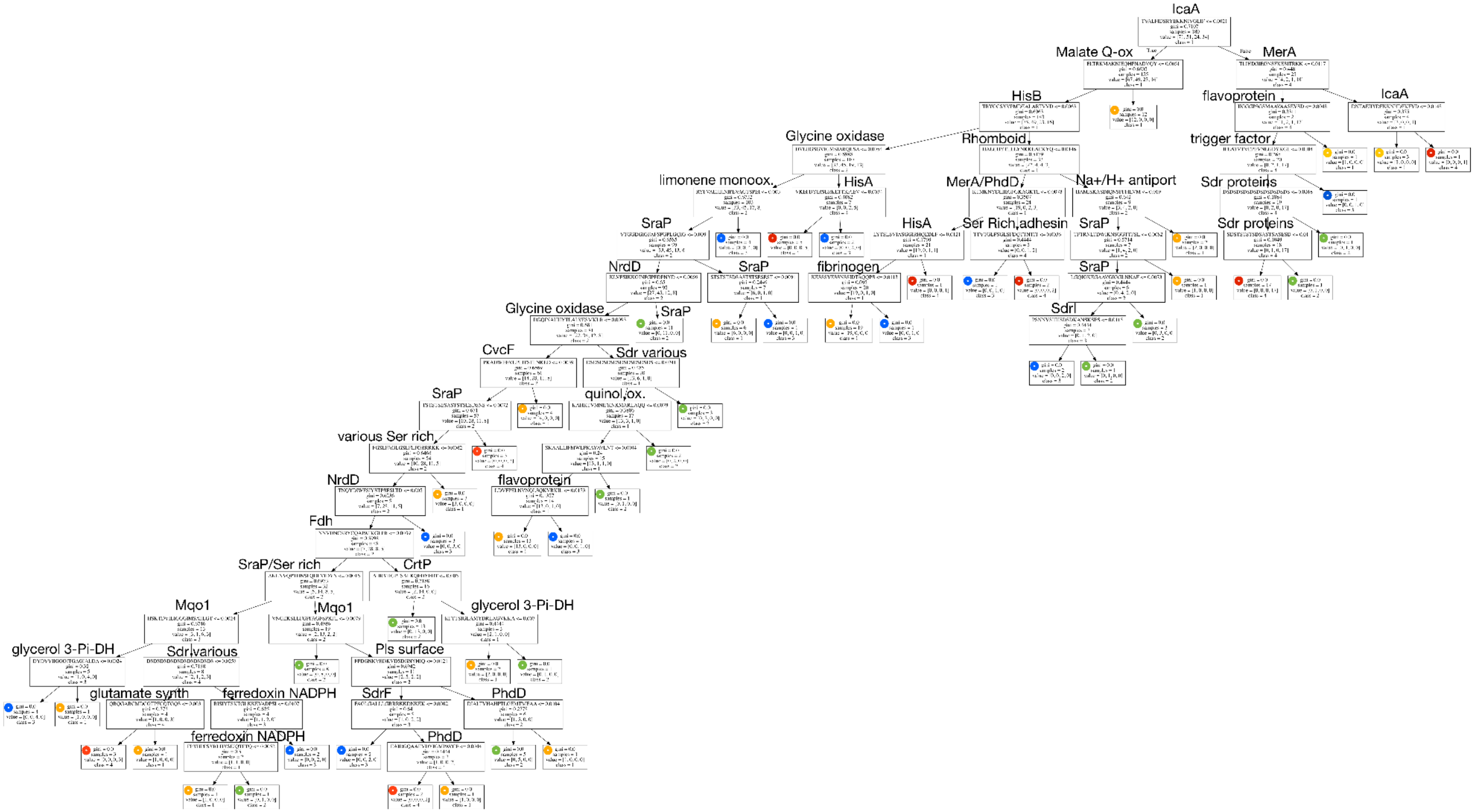

Composition of Chemically Defined Medium (Hussain, Hastings, & White, 1991)

**Component**	**mg/L**	**Component**	**mg/L**
Solution 1:		*Solution 2:*	
$Na_2HPO_4.2H_2O$	10000	Glucose	10000
KH_2PO_4	3000	$MgSO_4.7H_2O$	500
L-Aspartic acid	150		
L-Alanine	100	*Solution 3:*	
L-Arginine	100	Biotin	0.1
L-Cystine	50	Nicotinic acid	2
Glycine	100	D-pantothenic acid, Ca salt	2
L-Glutamic acid	150	Pyridoxal	4
L-Histidine	100	Pyridoxamine dihydrochloride	4
L-Isoleucine	150	Riboflavin	2
L-Lysine	100	Thiamin hydrochloride	2
L-Leucine	150		
L-Methionine	100	*Solution 4:*	
L-Phenylalanine	100	Adenine sulphate	20
L-Proline	150	Guanine hydrochloride	20
L-Serine	100		
L-Threonine	150	*Solution 5:*	
L-Tryptophan	100	$CaCl_2.6H_2O$	10
L-Tyrosine	100	$MnSO_4$	5
L-Valine	150	$(NH_4)2SO_4.FeSO_4.6H_2O$	6

Medium production:

Add Solution 1 components to 700 mL water. Make concentrated stock solutions of Solution 3 (100x), Solution 4 (20x) and Solution 5 (100x). Mix all solutions except 2, add distilled water to 900 mL and autoclave to sterilise. Make Solution 2 in 100 mL water (10x), autoclave separately and add to the main medium when cool.

Supplementary Figure 2: Melt curves and validation graphs for RT-qPCR primers.

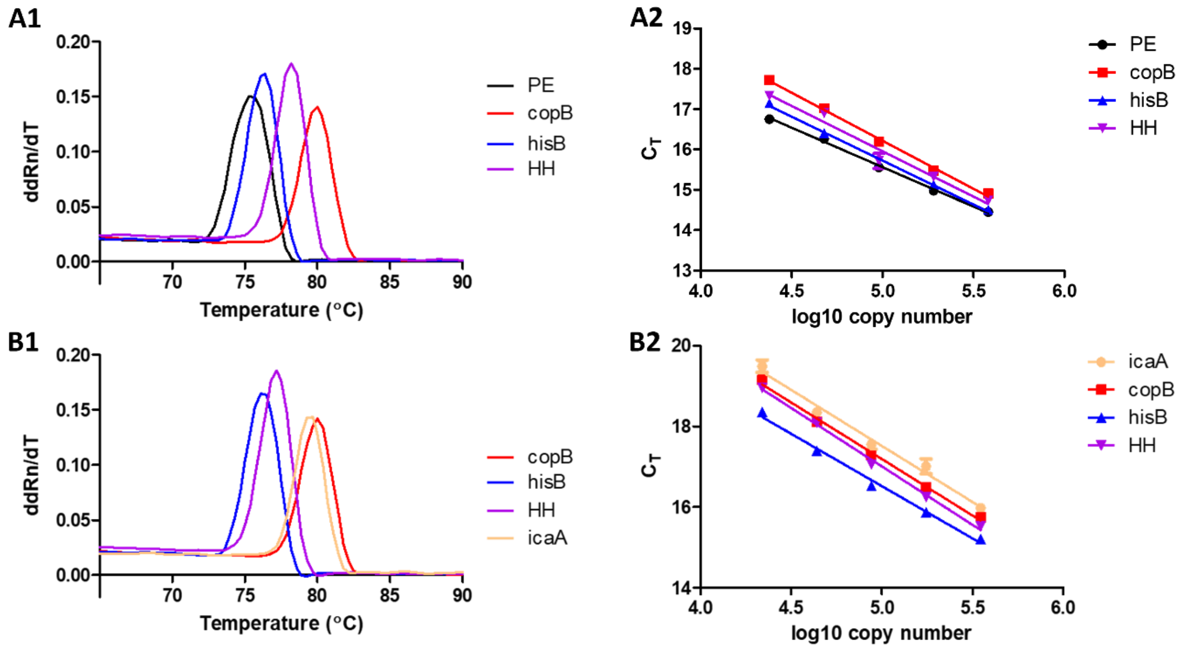

A) *Staphylococcus epidermidis* 846 gDNA used, B) *Staphylococcus epidermidis* RP62A gDNA used. 1) melt curves of RT-qPCR products showing clear single products, 2) CT values obtained across a range of DNA concentrations (each point is the mean of 3 replicates) and line of best fit. Primers were only used where lines of best fit were calculated to have an R^2 value above 0.95.

Supplementary Figure 3:

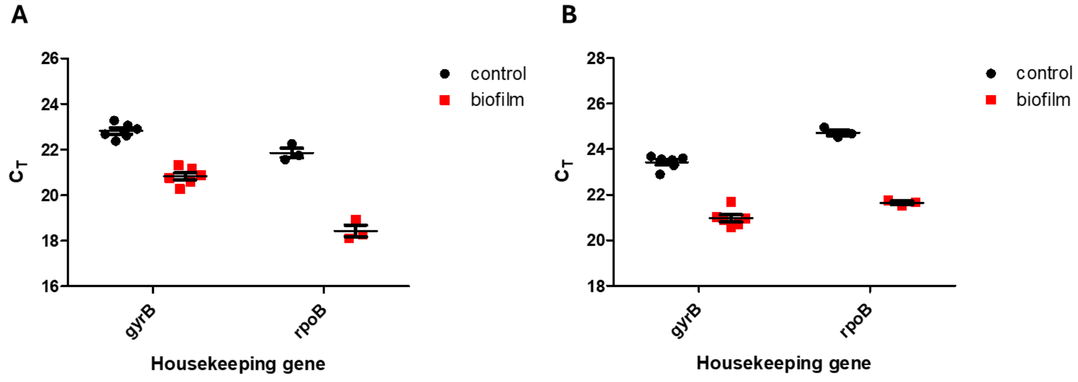

RT-qPCR C_T values for commonly used housekeeping genes *gyrB* and *rpoB* in RNA samples from planktonic and biofilm cultures of *Staphylococcus epidermidis* A) strain 846 and B) strain RP62A.

References:

Hussain, M., Hastings, J. G. M., & White, P. J. (1991). A chemically defined medium for slime production by coagulase-negative staphylococci. *Journal of Medical Microbiology*, *34*, 143-147.

Love, M. I., Huber, W., & Anders, S. (2014). Moderated estimation of fold change and dispersion for RNA-seq data with DESeq2. *Genome Biology*, *15*(12), 550. https://doi.org/10.1186/s13059-014-0550-8
